# A Novel Modified Electrode for Detection of the Food Colorant Sunset Yellow Based on Nanohybrid of MnO_2_ Nanorods-Decorated Electrochemically Reduced Graphene Oxide

**DOI:** 10.3390/molecules24061178

**Published:** 2019-03-25

**Authors:** Ziyu Ding, Peihong Deng, Yiyong Wu, Yaling Tian, Guangli Li, Jun Liu, Quanguo He

**Affiliations:** 1 School of Life Sciences and Chemistry, Hunan University of Technology, Zhuzhou 412007, China; dingziyu0320@163.com (Z.D.); wyy5082010@163.com (Y.W.); tianyaling0212@163.com (Y.T.); guangli010@hut.edu.cn (G.L.); liu.jun.1015@163.com (J.L.); 2 Key Laboratory of Functional Metal-Organic Compounds of Hunan Province, Key Laboratory of Functional Organometallic Materials of Hunan Provincial Universities, Department of Chemistry and Material Science, Hengyang Normal University, Hengyang 421008, China

**Keywords:** colorant analysis, sunset yellow, MnO_2_ nanorods, electrochemical reduced graphene oxide, voltammetric determination

## Abstract

The nanohybrid of electrochemically-reduced graphene oxide (ERGO) nanosheets decorated with MnO_2_ nanorods (MnO_2_ NRs) was modified on the surface of a glassy carbon electrode (GCE). Controlled potential reduction was applied for the reduction of graphene oxide (GO). The characterization was performed by scanning electron microscopy, X-ray diffraction and cyclic voltammetry. Compared with the poor electrochemical response at bare GCE, a well-defined oxidation peak of sunset yellow (SY) was observed at the MnO_2_ NRs-ERGO/GCE, which was attributed to the high accumulation efficiency as well as considerable electrocatalytic activity of ERGO and MnO_2_ NRs on the electrode surface. The experimental parameters for SY detection were optimized in detail. Under the optimized experiment conditions, the MnO_2_ NRs-ERGO/GCE showed good linear response to SY in concentration range of 0.01–2.0 μM, 2.0–10.0 μM and 10.0–100.0 μM with a detection limit of 2.0 nM. This developed method was applied for SY detection in soft drinks with satisfied detected results.

## 1. Introduction

Sunset Yellow (SY) is a water-soluble synthetic colorant, extensively used in the food industry because of its excellent color uniformity, low production cost, and high stability. However, the content of SY in foods must be strictly controlled and SY is not allowed to be added to fresh meat because it can cause allergies, diarrhea and other symptoms in sensitive people [1]. When the intake is too large, it will accumulate in the body and cause kidney and liver damage. When SY is used as food additive, the required content is less than 50 ppm [2]. Therefore, for food safety and human health it is quite important to develop a simple, rapid and sensitive method for the detection of SY.

At present, some analytical methods for SY detection have been reported, such as spectrophotometry [3], high performance liquid chromatography (HPLC) [4,5], HPLC-mass spectrometry (HPLC-MS) [6], capillary electrophoresis [7,8], and fluorescence emission spectrometry [9]. Spectrophotometry, capillary electrophoresis and fluorescence techniques either suffer from low sensitivity, narrow linear ranges or high detection limits. Although the chromatographic methods can offer good selectivity and detection limits, they often require time-consuming detection processes and complex pre-treatment steps. Moreover, these instruments are rather complicated, expensive, and cannot be employed for on-site measurements. Compared with the above methods, the newly developed electrochemical methods have received more attention in practical applications due to their advantages of simplicity, low cost, high sensitivity, and convenience for in-situ detection. Some chemically modified electrodes have been reported for the electrochemical detection of SY, For example, a cetyltrimethylammonium bromide-functionalized montmorillonite calcium-modified carbon paste electrode (CTAB-MMT-Ca/CPE) [10], a Au nanoparticles/graphene-modified glassy carbon electrode (Au-RGO/GCE) [11], a gold nanorods-decorated graphene oxide-modified glassy carbon electrode (AuNRs-GO/GCE) [12], a platinum nanoparticles-functionalized graphene composite-modified glassy carbon electrode (CTAB-Gr-Pt/GCE) [13], a multi-walled carbon nanotubes and graphene oxide nanocomposite-modified glassy carbon electrode (GO/MWCNTs/GCE) [14], a ZnO/cysteic acid nanocomposite-modified glassy carbon electrode (ZnO/cysteic acid/GCE) [15], a bimetallic nanoparticle-functionalized graphene-modified glassy carbon electrode (PDDA-Gr-(Pd-Pt)/GCE; PDDA-Gr-(Pt-Cu)/GCE;PDDA-Gr-(Co-Ni)/GCE) [16], a chitosan/graphene-modified glassy carbon electrode (Chit-Gr/GCE) [17], etc. The performance of these modified electrodes is strongly dependent on the modified materials. Table 1 and Table 2 summarize the comparison and advantage data of the different modified electrodes in SY detection. Each approach has its particular sensitivity and is subject to various limitations. Therefore, it is still necessary to identify new materials to detect SY accurately and rapidly.

Many researchers have been studying nanoparticles for electrochemical sensors, especially transition metal oxide nanoparticles such as Fe_2_O_3_ [18], Fe_3_O_4_ [19,20], Cu_2_O [21,22], Co_3_O_4_ [23], TiO_2_ [24], NiO [25], etc,, which have become the most popular material due to their unique properties of low cost, large surface area, good biocompatibility and distinct catalytic activity. Among them, non-toxic, economical and effective MnO_2_ nanoparticles have been extensively developed [26,27,28,29]. They can be easily synthesized into various shapes, including rods, porous materials, plates, tubes, wires, spheres and many others [30,31,32,33,34,35,36,37]. The electrochemical properties of MnO_2_ nanoparticles can be easily adjusted by tailoring their shape or morphology [38]. MnO_2_ nanorods (MnO_2_ NRs) are tiny, rod-like nanoparticles which have many interesting functions based on their anisotropic shapes. However, the poor dispersibility and the poor conductivity of MnO_2_ NRs have limited their utility in electrochemical sensors. To overcome these drawbacks, intensive efforts have been applied toward coupling MnO_2_ with graphene (GR), because GR is an attractive electrode material with a high theoretical specific surface area (2520 m^2^/g) and a high electrical conductivity, and a good candidate as a carrier [39,40]. The incorporation of GR with MnO_2_ can produce synergistic effects leading to improved conductivity, enhanced catalytic activity and improved stability of the MnO_2_ nanoparticles. In previous studies, different crystalline forms and morphologies of the MnO_2_ nanoparticles were assembled for electrode decoration by using GR [41,42,43]. The synergistic effect is outstanding, which confers them a great potential to replace conventional catalysts. However, as for the preparation of such compounds, conventional methods usually require complex separation processes, including multiple filtration stages and high-speed centrifugation [44,45]. These are the main obstacles for practical applications. Therefore, the development of an effective method for preparing the MnO_2_–GR hybrid materials is of significant importance.

In our previous work, we proposed an electroreduction technology for the fabrication of MnO_2_–graphene hybrid materials with high efficiency and relatively low operating cost [46,47]. Graphene oxide (GO), a derivative of graphene, is highly hydrophilic and dispersible due to its large number of oxygen-containing functional groups. Therefore, a dispersion of GO and MnO_2_ NRs was dropped onto the surface of a glassy carbon electrode (GCE), and then electrochemical reduction of GO was carried out. The conductivity of electrochemical reduced GO (ERGO) is much higher than that of GO due to the recovery of the conductive carbon conjugated networks. We found that this hybrid material showed superior electrocatalytic activity toward amaranth [46] and dopamine [47]. However, sensitive and rapid detection of SY using this hybrid material has not been reported yet.

In the present study, a MnO_2_ NRs-ERGO nanocomposite-modified GCE (denoted as MnO_2_ NRs-ERGO/GCE) has been prepared by a facile method. The morphology of the nanocomposite was investigated by scanning electron microscopy (SEM) and X-ray diffraction (XRD), and the electrochemical behaviour of the modified electrode was studied by cyclic voltammetry (CV) and second-order derivative linear sweep voltammetry (SDLSV). Due to electrocatalytic activity of MnO_2_ NRs-ErGO/GCE toward SY oxidation, a novel electrochemical sensing platform for SY was developed. The analytical characteristics of the sensor were studied in detail and its applicability toward SY detection in real samples was evaluated.

## 2. Results

### 2.1. Characteristics of the Nanohybrid

**Figure 1:**
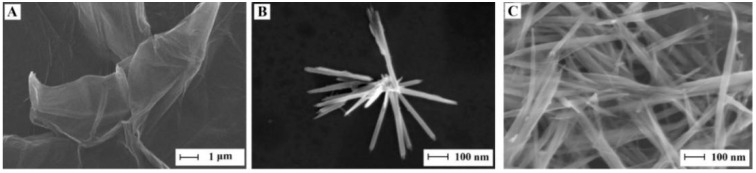
SEM images of GO (**A**), MnO_2_ NRs (**B**), and MnO_2_ NRs-ERGO (**C**).

The morphology of the materials was revealed by SEM studies. Wrinkled, aggregated, and thin sheets of GO can be observed in Figure 1A. As seen in Figure 1B, the MnO_2_ NRs had a uniform nanorods-like structure (~44 nm in diameter and ~800 nm in length on an average). In Figure 1C, the MnO_2_ NRs are randomly assembled with the ERGO flakes. The ERGO flakes were self-assembled in a layered structure with MnO_2_ NRs embedded between the layers, suggesting the MnO_2_ NRs were combined with ERGO successfully.

**Figure 2:**
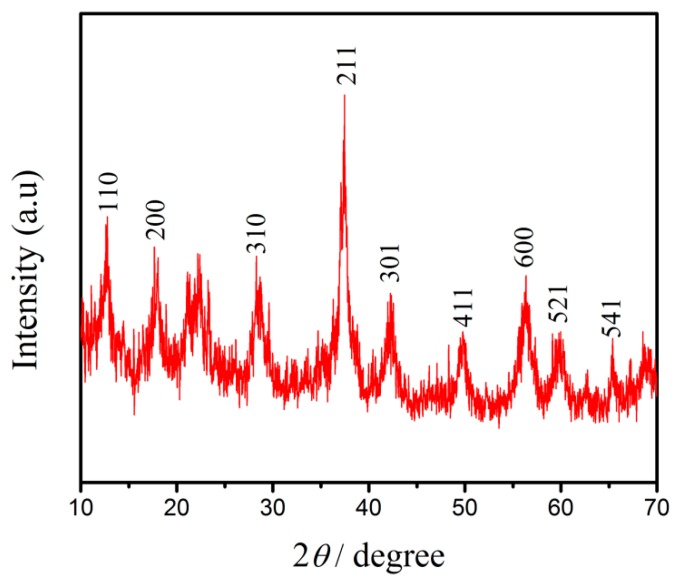
XRD pattern of MnO_2_ NRs.

Figure 2 illustrates the XRD pattern of the MnO_2_ NRs recorded in the 2θ range of 10–70°. It was observed that the characteristic reflections of the MnO_2_ NRs were shown at 2θ = 12.1°, 18.0°, 29.3°, 37.5°, 42.1°, 50.1°, 56.5°, 60.5°, and 69.8°, corresponding to the lattice planes of (110), (200), (310), (211), (301), (411), (600), (521) and (541), which were well coincided with the standard data file (JSPDS 44-0141), suggesting α-MnO_2_was perfectly crystallized.

### 2.2. Electrochemical Behaviors of SY at Different Electrodes

**Figure 3:**
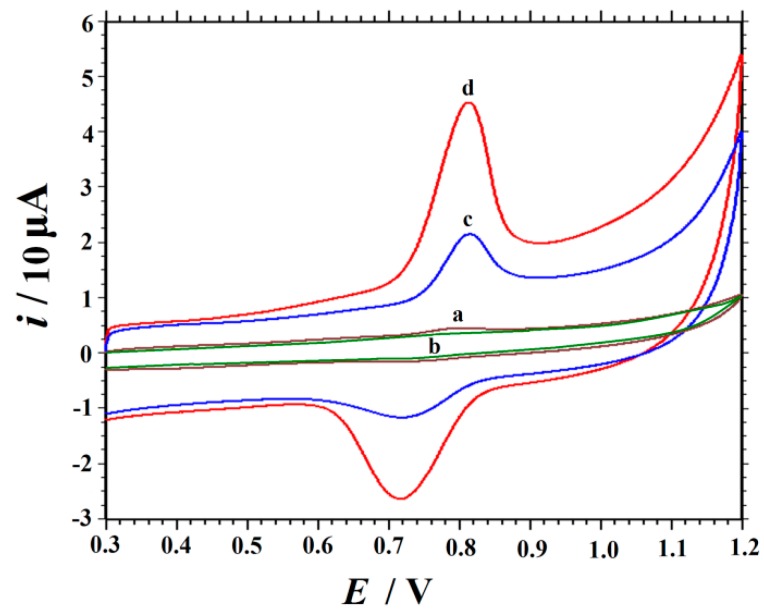
Cyclic voltammograms of 0.1 mM SY recorded at GCE (**a**), GO/GCE (**b**), ERGO/GCE (**c**) and MnO_2_ NRs-ERGO/GCE (**d**) in 0.3 M citric acid-sodium citrate buffer (pH 4.5), scan rate 0.1 V/s.

The cyclic voltammograms (CVs) of 0.1 mM SY in 0.3 M citric acid-sodium citrate buffer (pH = 4.5) solution at different modified electrodes within the potential range from 0.3 to 1.2 V at a scan rate of 0.1 V/s are exhibited in Figure 3, where it can be seen that there is a very small oxidation peak (*E*_pa_ = 0.804 V, *i*_pa_ = 1.693 μA) of SY on bare GCE, indicating a slow electron transfer kinetic. At GO/GCE, the oxidation peak current of SY was smaller than that of GCE because of the low conductivity of GO. At ERGO/GCE, an improved oxidation peak (*i*_pa_ = 23.34 μA) at 0.816 V and a greatly enhanced reduction peak (*i*_pc_ = 10.88 μA) at 0.717 V were exhibited, indicating that ERGO was favorable for the electrocatalysis of SY. After ERGO was decorated with MnO_2_ NRs, a pair of well-defined redox peaks located at 0.814 V and 0.716 V appeared at the MnO_2_ NRs-ERGO/GCE. This pair of quasi-reversible peaks had stronger current responses (*i*_pa_ = 61.73 μA, *i*_pc_ = 35.48 μA) than the abovementioned electrodes. The oxidation peak current was 2.6, 50.6, and 36.5-fold those at ERGO/GCE, GO/GCE, and bare GCE, respectively. These results proved that MnO_2_ NRs-ERGO could readily facilitate electron transfer. MnO_2_ NRs has excellent electrocatalytic activity, which can be used as an electronic mediator to promote the transfer of electrons between the electrode and SY. From the SEM image B in Figure 1, it can be seen that regular high purity nanorods provide good crystallization, which is favorable for reducing the probability of the recombination of electrons and thus reduces the chemical energy barrier. Additionally, the nanorods–like MnO_2_ in Figure 1C show good dispensability and no obvious agglomeration is observed, plus the significantly rough surfaces and abundant pores, so the specific surface area of MnO_2_ NRs-ERGO composite increases dramatically. It is well known that large specific surface areas provide more active sites and absorb more analytes. Moreover, these pores also allow the electrons to transit inside their interior pore channels, which would improve electrocatalytic activity [38]. ERGO has good conductivity and high specific surface area. Furthermore, the remained O-H functional groups on ERGO also act as catalytic active sites and contribute to the oxidation of SY [48], thereby improve the performance of the modified electrode.

**Figure 4:**
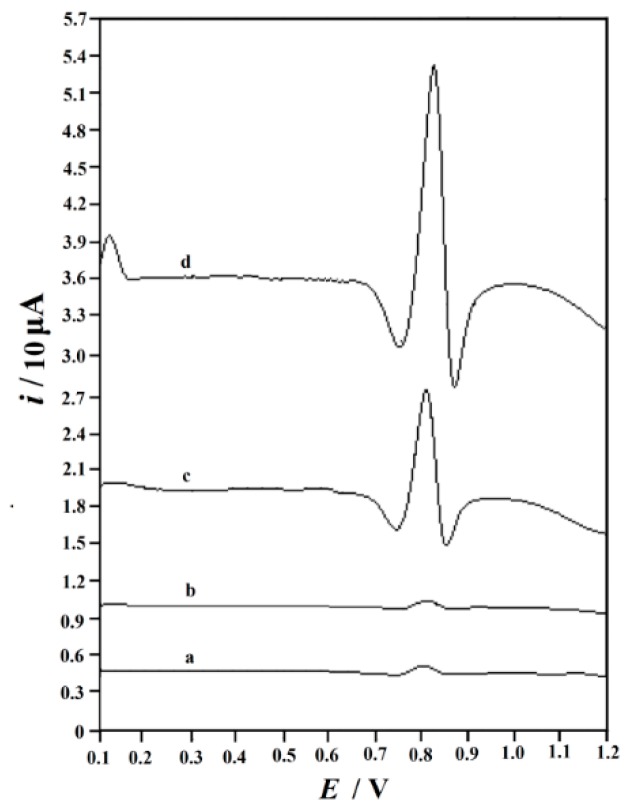
Second-order derivative linear scan voltammograms of 0.1 mM SY recorded at GCE (**a**), GO/GCE (**b**), ERGO/GCE (**c**) and MnO_2_ NRs-ERGO/GCE (**d**) in 0.3 M citric acid-sodium citrate buffer (pH 4.5). Accumulation potential: 0.1 V, accumulation time: 180 s, scan rate 0.1 V/s.

The electrochemical behavior of SY on the surface of GCE, GO/GCE, ERGO/GCE, and MnO_2_ NRs-ERGO/GCE was also studied using second derivative linear sweep voltammetry (SDLSV), and the results are shown in Figure 4. On the surface of GCE (curve a), the oxidation peak of SY was very weak (*i*_pa_ = 1.537 μA). When using the GO/GCE (curve b), the oxidation peak current of SY decreased slightly (*i*_pa_ = 1.244 μA). However, the oxidation peak of SY at 0.816 V was enhanced significantly (*i*_pa_ = 24.30 μA) on the surface of ERGO/GCE (curve c), indicating the superiority of ERGO due to its good conductivity, big surface area, and electrocatalytic ability towards SY. While on MnO_2_ NRs-ERGO/GCE the biggest peak current of 60.08 μA appeared at 0.814 V (curve d). The remarkable peak current enlargement revealed that MnO_2_ NRs-ERGO/GCE exhibited strong signal enhancement toward the oxidation of SY. From the comparison, we clearly found that MnO_2_ NRs-ERGO facilitated the oxidation of SY, and was more sensitive for SY detection.

### 2.3. Effect of Scan Rate

**Figure 5:**
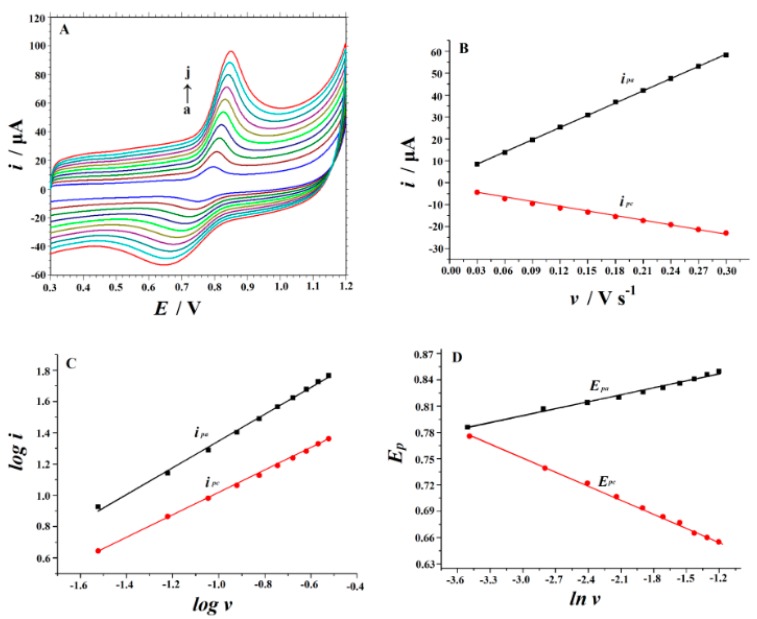
(**A**) Cyclic voltammograms of 10 μM SY at MnO_2_ NRs-ERGO/GCE with different scan rates. Curves (a–j) are obtained at 30, 60, 90, 120, 150, 180, 210, 240, 270 and 300 mV/s, respectively. (**B**) The plot for the dependence of peak current on scan rate. (**C**) The relationship between log *i*_p_ and log *v*. (**D**) The relationship between *E*_p_ and ln *v*.

In order to investigate the reaction kinetics of SY on the MnO_2_ NRs-ERGO/GCE, cyclic voltammograms with different scan rates were recorded (Figure 5A). As shown in Figure 5B, the anodic peak current (i_pa_) and cathodic peak current (i_pc_) of SY were linearly proportional to the scan rate (v) ranging from 0.03 to 0.3 V/s. The linear equations were as follows, indicating that the electrochemical process of SY is mainly controlled by adsorption:

*i*_pa_ (μA) = 186.04*v* (V s^−1^) + 2.9024 (R^2^ = 0.9998) (1)

*i*_pc_ (μA) = −67.257*v* (V s^−1^) −3.1713 (R^2^ = 0.996) (2)

Figure 5C illustrates the relationships between log *i* vs. log *v*. The corresponding equations can be expressed as follows:

log *i*_pa_ (μA) = 0.855 log *v* (V s^−1^) +2.2022 (R^2^ = 0.997) (3)

log *i*_pc_ (μA) = 0.7101 log *v* (V s^−1^) +1.7241 (R^2^ = 0.9990) (4)

The slopes obtained were 0.855 and 0.7101 (approximately equal to 1), confirming the adsorption-controlled nature of the electrode process of SY. Meanwhile, as depicted in Figure 5D, the anodic peak potentials (E_pa_) and cathodic peak potentials (E_pc_) of SY are linearly related to the Napierian logarithm of scan rate (ln *v*) in the range of 0.03–0.3 V/s. The equations are found to be:

*E*_pa_ (V) = 0.0314 ln *v* (V/s) + 0.8864 (R^2^ = 0.996) (5)

*E*_pc_ (V) = −0.0517 ln *v* (V/s) +0.5957 (R^2^ =0.990) (6)

Based on Laviron’s model [49], the slopes of the line for *E*_pa_ and *E*_pc_ can be expressed as RT/(1–α) nF and RT/αnF, respectively. Therefore, the values of the electron-transfer coefficient (α) and the electron-transfer number (*n*) can be calculated to be 0.38 and 1.31, respectively.

### 2.4. Effect of Buffer pH

**Figure 6:**
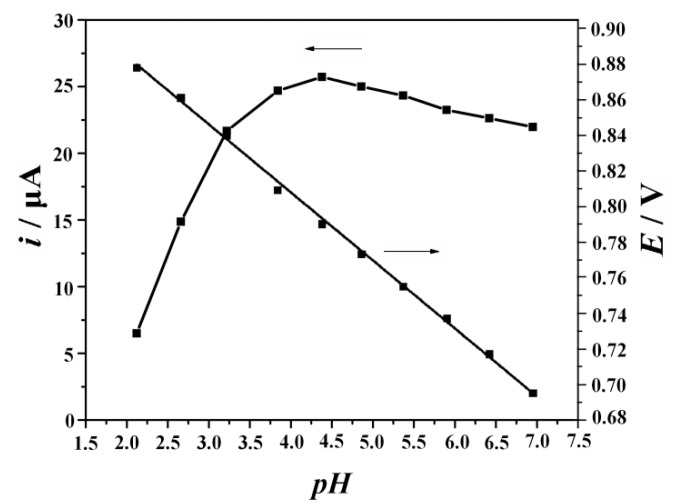
Effects of pH on the current response and potential response of 10 μM SY. Accumulation potential: 0.1 V, accumulation time: 180 s, scan rate 0.1 V/s.

The electrochemical response of SY on the MnO_2_ NRs-ERGO/GCE was investigated in 0.3 M citric acid-sodium citrate buffer at different pH values ranging from 2.0 to 8.0. As can be seen from Figure 6, the maximum oxidation peak current was obtained at pH 4.5 and it decreased gradually with the further increase of the pH value. Therefore, in the following experiments, pH 4.5 was chosen as the optimal pH value for SY determination. At the same time, the peak potential was found to be shifted negatively with the increase of buffer pH, indicating that proton participate in the electrochemical reaction. A linear regression equation was obtained as:

*E*_pa_ (V) = −0.0481 pH + 0.9972 (R^2^ = 0.9992) (7)

**Scheme 1:**
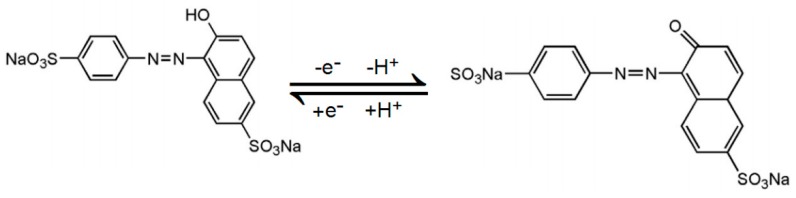
The electrode reaction mechanism for SY on the MnO_2_ NRs-ERGO/GCE.

The slope of −0.0481 was close to the theoretical value of −0.059 V/pH, indicating that the number of electrons involved in SY oxidation is equal to the number of protons. According to the above results, the electrooxidation of SY on MnO_2_ NRs-ERGO/GCE was a one-electron one-proton process. The mechanism of its electrochemical process can be expressed as Scheme 1.

### 2.5. Effect of Accumulation Conditions

Because the oxidation of SY on MnO_2_ NRs-ERGO/GCE is controlled by adsorption, the influence of accumulation conditions cannot be ignored. The effect of the accumulation potential and accumulation time on the oxidation current of 10 μM SY was investigated. It was revealed that when the accumulation potential shifted from −0.30 to 0.30 V, the current of SY changed slightly. Consequently, accumulation was carried out at the initial potential. The effect of accumulation time on the currents of SY was also investigated. The current increased significantly with the prolongation of accumulation time from 0 to 180 s. However, when the accumulation time exceeded 180 s, the current increased slowly, which indicated that the adsorption of SY on the electrode surface was supersaturated. Therefore, the accumulation time of 180 s was selected to determine SY.

### 2.6. Chronocoulometry

According to the expression given by Anson [50], the electrochemical effective surface areas of bare GCE and MnO_2_ NRs-ERGO/GCE can be obtained by chronocoulometry:

Q = 2nFAcD^1/2^π^−1/2^t^1/2^ + Q_dl_ + Q_ads_ (8)

**Figure 7:**
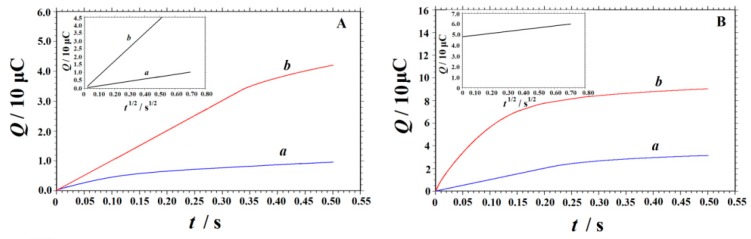
(**A**) Plot of Q–t curves of GCE (**a**) and MnO_2_ NRs-ERGO/GCE (**b**) in 1.0 mM K_3_[Fe(CN)_6_] containing 1.0 M KCl. Insert: Plot of Q–t^1/2^ curves on GCE (**a**) and MnO_2_ NRs-ERGO/GCE (**b**). (**B**) Plot of Q–t curves of the MnO_2_ NRs-ERGO/GCE in 0.3 M citric acid-sodium citrate buffer (pH 4.5) in the absence (**a**) and presence (**b**) of 0.1 mM SY. Insert: Plot of Q–t ^1/2^ curve on MnO_2_ NRs-ERGO/GCE after background subtracted.

In the formula, A is the surface area of the working electrode, *c* is the substrate concentration, D is the diffusion coefficient, Q_dl_ is the double layer charge, which can be eliminated by background subtraction, Q_ads_ is the adsorption charge. This experiment was performed in 1.0 mM K_3_[Fe(CN)_6_] solution containing 1.0 M KCl, where the diffusion coefficient of K_3_[Fe(CN)_6_] is 7.6 × 10^−6^ cm^2^ s^−1^ [51]. According to the experiment results (shown in Figure 7A), A was calculated to be 0.061 cm^2^ and 0.293 cm^2^ for GCE and MnO_2_ NRs-ERGO/GCE, respectively. These results showed that the effective surface area of the modified electrode increased obviously, which would improve the current response and decrease the detection limit.

The electrooxidation of SY at the MnO_2_ NRs-ERGO/GCE was also studied by chronocoulometry. The corresponding chronocoulometric curves are displayed in Figure 7B. The diffusion coefficient *D* and the adsorption charge Q_ads_ can be determined by Equation (8). As shown in the insert of Figure 7B, the relationship between Q and t^1/2^ was shown as a straight line after background subtraction. The slope was 1.652 × 10^−^^5^ C·s^−1/2^ and the intercept (Q_ads_) was 4.813 × 10^−^^5^ C. As n = 1, A = 0.293 cm^2^, and *c* = 0.1 mM, *D* was calculated to be 2.68 × 10^−^^5^ cm^2^·s^−1^. According to the equation Q_ads_ = nFAΓ_s_, the adsorption capacity Γ_s_ was 1.70 × 10^−^^9^ mol·cm^−2^. These results confirmed the remarkable enhancement effect of MnO_2_ NRs-ERGO for SY oxidation.

### 2.7. Analytical Properties

#### 2.7.1. Repeatability, Reproducibility and Stability

A solution containing 10 μM SY was used for the investigation of the repeatability, reproducibility and stability of MnO_2_ NRs-ERGO/GCE by SDLSV. Repetitive determinations were carried out on a single electrode. The used MnO_2_ NRs-ERGO/GCE could be regenerated easily by voltammetric sweeps between 0.0 V to 1.2 V in a blank solution. The relative standard deviation (RSD) for the peak currents of SY based on seven replicates was obtained as 2.56%. The reproducibility was studied by fabricating seven modified electrodes which were applied for SY detection, the result of RSD with 5.32% revealed the excellent reproducible of MnO_2_ NRs-ERGO/GCE. The stability of the MnO_2_ NRs-ERGO/GCE was studied over a two-week period by periodically measuring the peak currents of SY. The electrode remained 94.8% of its initial response value after two weeks, indicating that the MnO_2_ NRs-ERGO/GCE had acceptable storage stability.

#### 2.7.2. Interference Study

To evaluate the selectivity, the voltammetric response of 10 μM SY in the presence of different alien species were measured. The experimental data showed that no influences on the detection of 10 μM SY are found after addition of 1.0 mM Zn^2+^, Cu^2+^, Fe^3+^, Ca^2+^, Mg^2+^, Cl^−^, NO_3_^−^, SO_4_^2−^, CO_3_^2−^, glucose, oxalate, sucrose, glycine, alanine, L-cysteine, L-glutamine, L-serine, caffeine, benzoic acid; 0.5 mM vitamin C; 20 μM amaranth, allura red, brilliant blue, and 10 μM tartrazine, quinoline yellow (peak current change <10%). The results demonstrated that the MnO_2_ NRs-ERGO/GCE has a good selectivity for SY analysis in real samples.

#### 2.7.3. Calibration and Limit of Detection

**Figure 8:**
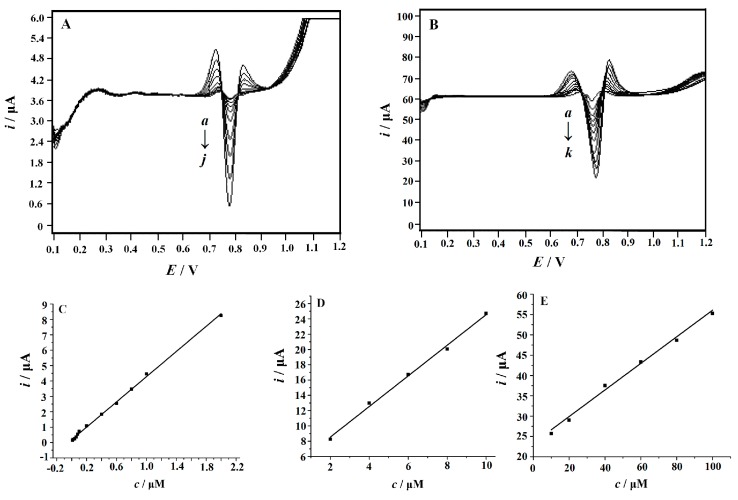
Second-order derivative linear scan voltammograms obtained at MnO_2_ NRs-ERGO/GCE in 0.3 M citric acid-sodium citrate buffer (pH 4.5) containing different concentrations of SY. (**A**) From a to j: 0.01, 0.02, 0.04, 0.06, 0.08, 0.1, 0.2, 0.4, 0.6, 0.8 μM; (**B**) From a to k: 1.0, 2.0, 4.0, 6.0, 8.0 10, 20, 40, 60, 80, 100 μM; (**C**–**E**) the calibration plots of the concentration of SY versus peak current (C: 0.01~2.0 μM; D:2.0~10 μM; E: 10~100 μM). Accumulation potential: 0.1 V, accumulation time: 180 s, scan rate 0.1 V/s.

Under the optimized experimental conditions, the quantitative analysis of SY was carried out by SDLSV. Figure 8 illustrates the SDLSV response of SY with different concentrations on MnO_2_ NRs-ERGO/GCE. A remarkable enhancement of peak current was observed with the increase of SY concentration. A good linearity was exhibited between the peak current of SY and its concentration in the range 0.01 μM~100 μM with three linear functions:

*i* (µA) = 4.0802*c* (µM) + 0.1832 (c = 0.01μM~2 μM) (R^2^ = 0.9983) (9)

*i* (µA) = 2.0014*c* (µM) + 4.5358 (c = 2 μM~10 μM) (R^2^ = 0.9965) (10)

*i* (µA) = 0.326*c* (µM) + 23.086 (c = 10 μM~100 μM) (R^2^ = 0.9944) (11)

The limit of detection (LOD) was estimated to be 2.0 nM (S/N = 3). As shown in Table 1 and Table 2, the performance of MnO_2_ NRs-ERGO/GCE is comparable to or superior to that of the previously reported modified electrodes [10,11,12,13,14,15,16,17]. In addition, this method has made remarkable improvements in simplifying the preparation of electrode, reducing cost and saving time, which proved that the electrode have good analytical performance and can be used for SY detection in real samples.

### 2.8. Practical Applications

The practical application of MnO_2_ NRs-ERGO/GCE for SY determination in real samples was testified in soft drinks with different China’s famous brands (Unified Xiangchenduo, Huiyuan Juice, Wahaha, Farmer’s Orchard, China). Before analysis by SDLSV, the samples were filtered to remove any suspended solids. The concentration of SY was obtained by the standard addition method. The results are listed in Table 3, where the contents of SY can be found to be 4.24~8.37 μM, and the recoveries were between 97.7% and 102.8%. In addition, the contents of SY were determined by high performance liquid chromatography (HPLC) to verify the accuracy of the new method. The results showed that the results obtained by HPLC and MnO_2_ NRs-ERGO/GCE were consistent, which indicates that the new method is accurate and feasible.

## 3. Experimental

### 3.1. Chemicals and Solutions

Potassium permanganate (KMnO_4_), graphite powder, manganese sulfate (MnSO_4_) were provided by Sinopharm Chemical Reagent Co., Ltd. (Shanghai, China). Sunset yellow (SY) was supplied by Aladdin (Shanghai, China). All analytical grade reagents were used as received without further purification. 0.04524 g of SY was dissolved in 100.00 mL deionized water to prepare a 1.0 mM standard stock solution. A series of low concentration working solutions were prepared by further dilution of the stock solution with water. 0.3 M citric acid-sodium citrate buffer with a pH of 4.5 was used as supporting electrolyte.

### 3.2. Instruments

The characterization was implemented on a Hitachi S-4800 scanning electron microscope (Hitachi, Tokyo, Japan) at an accelerating voltage of 30 kV and a powder X-ray diffractometer (PANalytical, Amsterdam, The Netherlands) with Cu Kα radiation (0.1542 nm). Cyclic Voltammetry (CV) was finished on a CHI 660E electrochemical workstation (Chenhua Corp. Shanghai, China). Second derivative linear sweep voltammetry (SDLSV) was carried out on a JP-303E polarographic analyzer (Chengdu Instrument Factory, Chengdu, China). A traditional three-electrode system for all electrochemical experiments was composed of a bare or modified glassy carbon electrode as working electrode, a platinum wire as auxiliary electrode and a saturated calomel electrode (SCE) as reference electrode. A pH-3c exact digital pH meter (Shanghai Leichi Instrument Factory, Shanghai, China) was used for solution pH measurements.

### 3.3. Preparation of GO-MnO_2_ NRs Nanocomposites

MnO_2_ NRs was synthesized by a hydrothermal method according to Gan et al. [38]. MnO_2_ NRs dispersions (1.0 mg/mL) were obtained by addition of MnO_2_ NRs (10 mg) to deionized water (10 mL) and ultrasonication for 1 h. Graphite oxide was prepared using a modified Hummer’s method according to our previous report [21]. GO was then exfoliated by dispersing GO (20 mg) in deionized water (20 mL), followed by ultrasonication treatment for 2 h. Afterwards, it was centrifuged at 6000 rpm for 30 min in order to remove the unexfoliated graphite oxide and unoxidized graphite. Then MnO_2_ NRs dispersion (5.0 mL, 1.0 mg/mL) was very slowly dropped into GO aqueous solution (5.0 mL) and ultrasonically dispersed for 2 h. A homogeneous black dispersion was obtained 

### 3.4. Electrode Fabrication

Before modification, the GCE with a diameter of 3 mm was polished on silk with 0.05 μM of α-Al_2_O_3_ slurry. After that, it was washed thoroughly with deionized water and cleared in anhydrous ethanol and deionized water in an ultrasonic bath. 5.0 μL of the obtained MnO_2_ NRs-GO dispersion was coated on the GCE surface and dried under an infrared lamp, followed by electrochemically reduction at a constant potential of −1.2 V for 120 s in a phosphate buffer solution (pH 6.5). The obtained modified electrode was denoted as MnO_2_ NRs-ERGO/GCE. For comparison, GO/GCE and ERGO/GCE were also prepared by the similar way.

## 4. Conclusions

This study provides a simple and practical method for preparing the nanohybrid of electrochemical reduced graphene oxide decorated with manganese dioxide nanorods (MnO_2_ NRs-ERGO), and the MnO_2_ NRs-ERGO-modified GCE exhibited superior electrocatalytic ability towards the oxidation of SY, which can be attributed to the strong catalytic activity of MnO_2_ NRs, high adsorption capacity and excellent conductivity of ERGO. The developed modified electrode exhibited excellent analytical performance such as fast response, low cost, high sensitivity and selectivity, as well as wide linear range and low detection limit for SY detection.

## Figures and Tables

**Table 1:** Performance comparison of different modified electrodes for SY detection.

Modified Electrode	Sensitivity (μA/μM)	Repeatability (RSD%)	Reproducibility (RSD%)	Stability	Interferences	Recovery (%)	References
CTAB-MMT-Ca/CPE	20.31	poor repeatability	3.9	-	1 mM vitamin C, glucose, glycine, citric acid, benzoic acid; 1 μM Tartrazine, quinoline yellow; 5 μM sudan red, amaranth had no interference	-	[10]
Au-RGO/GCE	0.496	2.56	5.32	20 days	0.5 mM of NaCl, MgCl_2_, NaNO_3_, Fe (NO_3_)_3_, glucose, tartrazine and new coccine had no interference	99.24–101.94	[11]
ERGO-AuNRs/GCE	0.0334	3.5	8.1	21 days	60 μM Zn^2+^, Cu^2+^, Mg^2+^, Ca^2+^, Fe^3+^, Cl^−^, NO_3_^−^, H_2_PO_4_^−^, HCO_3_^−^, HPO_4_^2−^, CO_3_^2−^; 12 μM glucose, saccharin, sucrose, glycine, citric acid, ascorbic acid; 6 μM quinoline yellow; ponceau 4R had no interference	89.4–108.8	[12]
CTAB-Gr-Pt/GCE	2.5481	-	-	-	1.0 mM citric acid, benzoic acid, glucose; 0.2 mM tartrazine, amaranth, allura red had no interference	96.25–98.25	[13]
GO/MWCNTs/GCE	0.4636	3.7	-	30 days	0.1 mM Cu^2+^, Zn^2+^, Na^+^, Cl^−^, K^+^, Mg^2+^, SO_4_^2−^, Ca^2+^, CO_3_^2−^, NH_4_^+^, NO_3_^−^; 10 μM uric acid, urea, glucose, oxalate, glycine, alanine, L-cysteine, L-tyrosine, L-glutamine, L-serine, valine had no interference	101.5–104.0	[14]
ZnO/Cysteic acid/GCE	2.81	2.55	4.46	30 days	1.0 mM NH_4_^+^, Ca^2+^, Fe^3+^, Al^3+^, Zn^2+^, Mn^2+^, Mg^2+^, Br^2212^, CO_3_^2−^, SO_4_^2−^, 0.2 mM starch, sucrose, glucose, uric acid, vitamin B_2_, vitamin B_6_, ascorbic acid, dopamine, citric acid; 20 μM amaranth, allura red and quinolone yellow had no interference	95.7–101.3	[15]
PDDA-Gr-(Pd-Pt)/GCE, PDDA-Gr-(Pt-Cu)/GCE, PDDA-Gr-(Co-Ni)/GCE	-	-	-	-	5.0 mM Mg^2+^, K^+^, Ca^2+^, Zn^2+^, Cl^−^, SO_4_^2−^, NO_3_^−^; 0.5 mM citric acid, glucose, ascorbic acid; 0.01 mM allura red, amaranth had no interference	95.3–103	[16]
Chit-Gr/GCE	0.018	3.5	-	-	1.0 μM citric acid and ascorbic acid had no interference	92.65–97.00	[17]
MnO_2_ NRs-ERGO/GCE	4.0802	2.56	5.32	14 days	1.0 mM Zn^2+^, Cu^2+^, Fe^3+^, Ca^2+^, Mg^2+^, Cl^−^, NO_3_^−^, SO_4_^2−^, CO_3_^2−^, glucose, oxalate, sucrose, glycine, alanine, L-cysteine, L-glutamine, L-serine, caffeine, benzoic acid; 0.5 mM vitamin C; 20 μM amaranth, allura red, brilliant blue, and 10 μM tartrazine, quinoline yellow	97.7–102.8	This work

**Table 2:** Comparison of the linear range and detection limit with other modified electrodes for the determination of SY.

Modified Electrodes	Technique	Supporting Electrolyte	Linear Range/μM	Correlation Coefficient	Detection Limit/μM	References
CTAB-MMT-Ca/CPE	^i^ DPV	0.1 M acetate buffer (pH 4.0)	0.0025 to 0.2	0.995	0.00071	[10]
Au-RGO/GCE	DPV	0.1 M PBS buffer (pH 4.0)	0.002–2.145	0.993	0.002	[11]
			2.145–109.145	0.994		
AuNRs-GO/GCE	DPV	0.1 M PBS (pH 6.0)	0.01–3.0	0.995	0.0024	[12]
CTAB-Gr-Pt/GCE	DPV	0.1 M PBS (pH3.0)	0.08–10.0	0.9984	0.0042	[13]
GO/MWCNTs/GCE	^j^ LSV	0.1 M PBS buffer (pH 5.0)	0.09–8.0	0.9982	0.025	[14]
ZnO/Cysteic acid/GCE	DPV	0.1 M PBS buffer (pH 5.0)	0.1–3.0	0.9977	0.03	[15]
PDDA-Gr-(Pd-Pt)/GCE	DPV	0.1 M PBS buffer (pH 3.0)	0.02–10.0	-	0.006	[16]
PDDA-Gr-(Pt-Cu)/GCE			0.02–10.0		0.004	
PDDA-Gr-(Co-Ni)/GCE			0.008–10.0		0.002	
Chit-Gr/GCE	CV	0.1 M PBS buffer (pH 6.0)	0.2–100	0.99	0.0666	[17]
MnO_2_ NRs-ERGO/GCE	SDLSV	0.3 M citrate buffer (pH 4.5)	0.01–2	0.9983	0.002	This work
			2–10	0.9965		
			10–100	0.9944		

**Table 3:** Determination of SY in beverage samples (*n* = 4).

Sample ^a^	Found ^b^/μM	Added/μM	Total Found ^b^/μM	Recovery/%	Content Determined by HPLC ^b^/μM
unified xiangchenduo	4.24 (±0.16)	4.00	8.35 (±0.03)	102.8	4.28 (±0.18)
huiyuan juice	6.28 (±0.31)	6.00	12.14 (±0.11)	97.7	6.17 (±0.34)
wahaha	8.37 (±0.37)	8.00	16.28 (±0.17)	98.9	8.45 (±0.46)
farmer’s orchard	5.65 (±0.23)	5.00	10.76 (±0.47)	101.0	5.52 (±0.24)

^a^ All samples were collected from local supermarkets. ^b^ Average ± confidence interval, the confidence level is 95%.
